# Platelet-rich plasma activates AKT signaling to promote wound healing in a mouse model of radiation-induced skin injury

**DOI:** 10.1186/s12967-019-2044-7

**Published:** 2019-08-28

**Authors:** Janet Lee, Hyosun Jang, Sunhoo Park, Hyunwook Myung, Kyuchang Kim, Hyewon Kim, Won-Suk Jang, Sun-Joo Lee, Jae Kyung Myung, Sehwan Shim

**Affiliations:** 1 Laboratory of Radiation Exposure & Therapeutics, National Radiation Emergency Medical Center, Korea Institute of Radiological and Medical Sciences, 75 Nowon-ro, Nowon-gu, Seoul, 01812 Republic of Korea; 2 Department of Pathology, Korea Cancer Center Hospital, Korea Institute of Radiological and Medical Sciences, Seoul, Republic of Korea

**Keywords:** Radiation, Keratin-14, Platelet-rich plasma, Regeneration, Skin

## Abstract

**Background:**

The skin is impacted by every form of external radiation therapy. However, effective therapeutic options for severe, acute radiation-induced skin reactions are limited. Although platelet-rich plasma (PRP) is known to improve cutaneous wound healing, its effects in the context of high-dose irradiation are still poorly understood.

**Methods:**

We investigated the regenerative functions of PRP by subjecting the dorsal skin of mice to local irradiation (40 Gy) and exposing HaCaT cells to gamma rays (5 Gy). The cutaneous benefits of PRP were gauged by wound size, histologic features, immunostains, western blot, and transepithelial water loss (TEWL). To assess the molecular effects of PRP on keratinocytes of healing radiation-induced wounds, we evaluated AKT signaling.

**Results:**

Heightened expression of keratin 14 (K14) was documented in irradiated HaCaT cells and skin tissue, although the healing capacity of injured HaCaT cells declined. By applying PRP, this capacity was restored via augmented AKT signaling. In our mouse model, PRP use achieved the following: (1) healing of desquamated skin, acutely injured by radiation; (2) activated AKT signaling, improving migration and proliferation of K14 cells; (3) greater expression of involucrin in keratin 10 cells and sebaceous glands; and (4) reduced TEWL, strengthening the cutaneous barrier function.

**Conclusions:**

Our findings indicate that PRP enhances the functions of K14 cells via AKT signaling, accelerating the regeneration of irradiated skin. These wound-healing benefits may have merit in a clinical setting.

## Background

The skin is vulnerable to every form of external radiation therapy administered to target internal organs [1]. Skin reactions following irradiation have characteristics of the delay in the onset of clinical changes. Acute skin reaction related to radiation therapy usually manifest within 1–4 weeks of radiation start. Even with modern radiotherapy techniques, ~ 85% of patients will experience moderate to severe acute skin reactions in exposed areas [2]. When severe, such exposures culminate in epidermal desquamation and potential ulceration or necrosis [3–5], thereby curtailing treatments and perhaps undermining cancer control or prognosis. At present, conventional postirradiation skin management [6] includes preventative measures, prompt conservative therapy, and even skin flaps in extreme cases. Still, conventional methods are less than ideal [7]. The key to managing irradiated skin is its regenerative potential.

Skin injury triggers immediate stress responses in epidermal keratinocytes, which then begin to proliferate and migrate to wounded areas, giving rise to a layer of hyperproliferative epithelium [8–11]. Epithelial tissues express differing keratin pairs, depending upon cell type. The K5/K14 pair is expressed by basal epidermis, wherein epidermal stem cells and transient amplifying (TA) cells reside [12]. It has been shown that K14 cells adjacent to wounds are intimately involved in epithelial regenerative processes, producing daughter cells that gravitate to sites of injury and assist in skin repair [12, 13]. However, cellular radiosensitivity is influenced by both phase of cellular proliferation and degree of differentiation, less differentiated or actively proliferating cells being more radiosensitive than highly differentiated or non-proliferating cells [14, 15]. Thus, impaired keratinocyte proliferation and differentiation in the aftermath of irradiation impedes skin regeneration and healing.

Platelet-rich plasma (PRP) has been incorporated into a wide variety of surgical procedures and clinical treatments. Especially in chronic wounds, PRP has shown promising experimental and clinical results [16]. The regenerative potential of PRP is generally attributed to supraphysiologic concentrations of various growth factors released by activated platelets [17, 18]. The essential roles of these growth factors in tissue regeneration and wound healing have been confirmed by many studies [19–21], particularly via PI3K/AKT/NFkappaB signaling pathways [22]. According to a recent publication, the PI3K/AKT pathway is canonical pathway to promote the keratinocyte proliferation [12]. Nevertheless, the ability of PRP to regulate postirradiation keratinocyte activity and its capacity to promote regeneration in irradiated skin is not fully understood. In the present study, we investigated the influence of radiation to the keratinocyte capacity and whether PRP enhances the regeneration efficacy of irradiated keratinocytes in vitro and in vivo in a mouse model of radiation-induced skin injury.

## Materials and methods

### PRP preparation

Umbilical cord blood (UCB) donated from ALLCORD (Seoul Metropolitan Government Public Cord Blood Bank) was used to prepare PRP, as detailed in a previously published protocol [18]. The plasma was first collected by centrifugation (250×*g*, 10 min). Platelets in collected plasma were then pelleted through a second centrifugation (1000×*g*, 10 min). Thereafter, PRP was activated by a bead mill homogenizer (Precellys 24; OMNI International, Kennesaw, GA, USA). The supernatants were collected by centrifugation (12,000×*g*, 20 min) and filtered (0.2-μm size) to furnish the activated PRP releasate.

### Cell culture

Human keratinocyte HaCaT cells and 293T cells were grown in Dulbecco’s Modified Eagle’s Media (DMEM; Gibco, Gaithersburg, MD, USA) containing 10% heat-inactivated fetal bovine serum (FBS; Gibco) and 1% antibiotic–antimycotic (Gibco) at 37 °C in a humidified atmosphere of 5% CO_2_. The cells were then irradiated at indicated dose using a ^137^Cs γ-ray source (Atomic Energy of Canada, Chalk River, ON, Canada) at a dose rate of 3.81 Gy/min. HaCaT cells were replaced to 0.5% FBS containing medium and added PRP or LY294002 (Sigma-Aldrich) for 24 h.

### Scratch assay

Cells seeded in 12-well plates were incubated for 24 h until confluent monolayers formed. Using a pipette tip to wound or scratch monolayers, each was then rinsed twice with PBS and incubated in fresh media for eventual fixation (4% formaldehyde) and staining (crystal violet). Under an inverted phase-contrast microscope, images were captured and measurements taken.

### RNA silencing and lentivirus replication

To generate K14 knockdown cells, a lentivirus-based plasmid kit (TR311838) encoding a 4-gene set (#1–#4) of unique 29mer shRNA sequences specific for the human K14 gene was purchased, along with non-effective control shRNA vectors (OriGene, Rockville, MD, USA). Lentivirus replication entailed transient co-transfection of HEK 293T cells with packaging vectors, using polyethylenimine (PEI) method. After 5 h, the medium was replaced with fresh; and 48 h later, the virus-laden medium was collected, cleared by centrifugation, and passed through 0.45-μm syringe filter.

HaCaT cells were infected with either K14 shRNA lentivirus (Lenti-K14 shRNA #1–#4) or negative control lentivirus (C) in the presence of 8 μg/ml polybrene (Sigma-Aldrich, St. Louis, MO, USA) for 16 h, and the medium was refreshed. After 3 days the infection, the efficiency of infection was measured by western blot analysis.

### Animal selection and care

Totally 72 male SKH-1 mice (7 weeks old, 30 ± 3 g) were obtained from the Orient Bio (Seongnam, South Korea). The mice were held under controlled conditions, including constant temperature, allowing free access to regular chow and 3-stage filtered water. After 1 week of acclimatization, mice were randomly divided into three groups. The Animal Investigation Committee of the Korea Institute of Radiological and Medical Sciences approved all animal experimentation.

### Irradiation and PRP treatment protocol

Animals were anesthetized by intraperitoneal injection of alfaxalone (Alfaxan, 75 mg/kg; Careside, Gyeonggi-do, Korea) and xylazine (Rompun, 10 mg/kg; Bayer Korea, Seoul, South Korea) for irradiation (X-RAD 320; Softex Korea, Gyeonggi-do, South Korea). The dorsal skin was gently stretched to required dimensions (2 cm × 2 cm) and taped, falling within the field of irradiation. Other bodily areas were protected by lead shielding (6 mm). A single dose of 40 Gy was delivered at 260 kV, 10 mA, using a rate of 2 Gy/min. Dosage and rate of delivery were strictly monitored (UNIDOS E Universal Dosemeter; PTW-Freiburg, Freiburg, Germany). Afterwards, topical PRP was applied twice weekly for 2 weeks by intradermal injection, for a total of 100 ul of PRP per injury site in treated mice.

### Transepidermal water loss in hairless mouse skin

Transepidermal water loss (TEWL) was quantified mechanically (Tewameter TM 300; Courage + Khazaka Electronic GmbH, Cologne, Germany) according to device protocol. Measurements were obtained under controlled conditions, including constant relative humidity and room temperature.

### Histologic examination of hairless mouse skin

All animals were sacrificed and the dorsal skin promptly excised. The excised patches (2 cm × 2 cm) were fixed in 4% paraformaldehyde, embedded in paraffin, sectioned at 4 μm, and stained [hematoxylin and eosin (H&E) and Sirius Red] for microscopic examination. Immunofluorescent stains were also performed to assess expression levels of K10, K14, Ki-67, and involucrin during wound healing. The following antibodies were purchased from commercial sources: anti-K10 (Abcam, Cambridge, UK), anti-K14 (Abcam), anti-Ki-67 (Acris Antibodies, Herford, Germany), and involucrin (Santa Cruz Biotechnology, Dallas, TX, USA). Quantitative assessment of immunoreactivity was enabled by proprietary image analysis software (i-Solution; IMT Inc, https://www.IMT-Solution.com).

### Whole-mount images of hairless mouse epidermis

Samples of promptly excised dorsal skin taken from sacrificed animals were incubated in 5 mM EDTA/PBS overnight at 4 °C, with an additional 2 h at 37 °C. Epidermis was smoothly peeled away using a pair of fine forceps and immediately placed in fresh PBS for Oil Red O staining of sebaceous glands.

### Western blot analysis

Cells or tissue specimens were homogenized in RIPA lysis and extraction buffer (Thermo Fisher Scientific, Waltham, MA, USA), separating lysates by electrophoresis in 12% sodium dodecyl sulfate-polyacrylamide gel. The various proteins were then electrophoretically transferred onto polyvinylidene fluoride membranes for blocking (5% skim milk, 1 h) and overnight incubation (4 °C) with the following primary antibodies: K1 (Abcam), K10 (Abcam), K14 (Abcam), Ki-67 (Acris Antibodies), p-AKT (Cell Signaling Technology, Danvers, MA, USA), AKT (Cell Signaling Technology), involucrin, and β-actin (Santa Cruz Biotechnology). Immunoreactive bands were developed using a horseradish peroxidase-linked secondary antibody (Santa Cruz Biotechnology) and visualized as directed by light emission (Western Lightning Plus-ECL Enhanced Chemiluminescence Substrate; PerkinElmer, Waltham, MA, USA). Quantitative assessment of immunoreactivity was software-enabled (IMT Inc).

### Statistical analysis

All data were subjected to Student’s *t*-tests and expressed as standard error of the mean (SEM). Statistical significance was set at *p *< 0.05.

## Results

### Irradiation (IR) altered expression patterns of keratins in vitro and in vivo

**Fig. 1:**
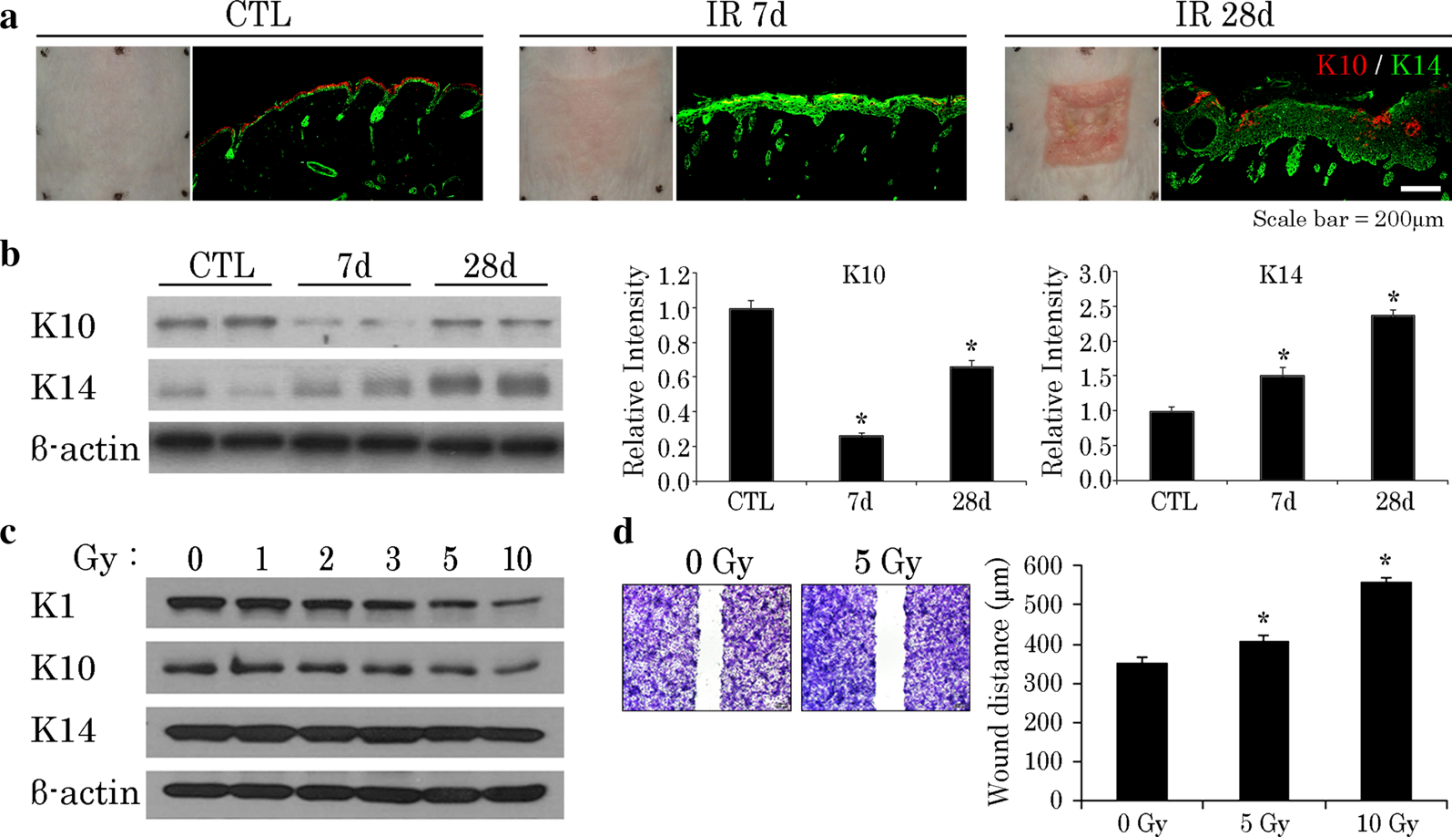
Irradiation (IR) modulates keratin expression patterns and suppresses wound-healing capacity of keratinocytes in mouse skin (40 Gy delivered locally). **a** Differing K10/K14 positivity on immunostain in normal and irradiated mouse skin. **b** Western blot analysis of K10 and K14 expression levels in normal and irradiated mouse skin, day 7, 28. Results of one experiment performed in triplicate and expressed as mean ± SEM *p < 0.05 vs. CTL group. **c** Expression levels of keratins 1, 10, and 14 in HaCaT cells irradiated at different doses, incubated for 24 h; and **d** wound-healing capacity of HaCaT cells irradiated at different doses, using scratch-simulated wound migration assay. Results of one experiment performed in triplicate and expressed as mean ± SEM (Student’s *t* test applied) *p < 0.05 vs. 0-Gy group. *CTL* control

Keratin filaments are critical in regulating cellular functions, serving to modulate various signaling pathways [23]. The paired K5/K14 keratins of proliferating basal epidermis gradually give way to K1/K10 as cells ascend during differentiation [12, 24]. Because cellular radiosensitivity reflects both phase of cellular proliferation and degree of differentiation [14], we anticipated that cells expressing K5/K14 keratins would prove more radiosensitive than those expressing K1/K10. Instead, our mouse model showed accumulation of K14 in irradiated skin tissues, rather than a decline; and K10 expression was deficient (Fig. 1a, b). To verify these observations, we subjected HaCaT cells to varying doses of irradiation (IR) as a dose–response trial. Subsequently, we found that IR effectively lowered K1/K10 expression and sustained K14 expression at doses > 5 Gy (Fig. 1c), implying a relation between radiation-induced skin injury and the functional status of K14-positive cells.

### IR delayed wound healing in HaCaT cells

**Fig. 2:**
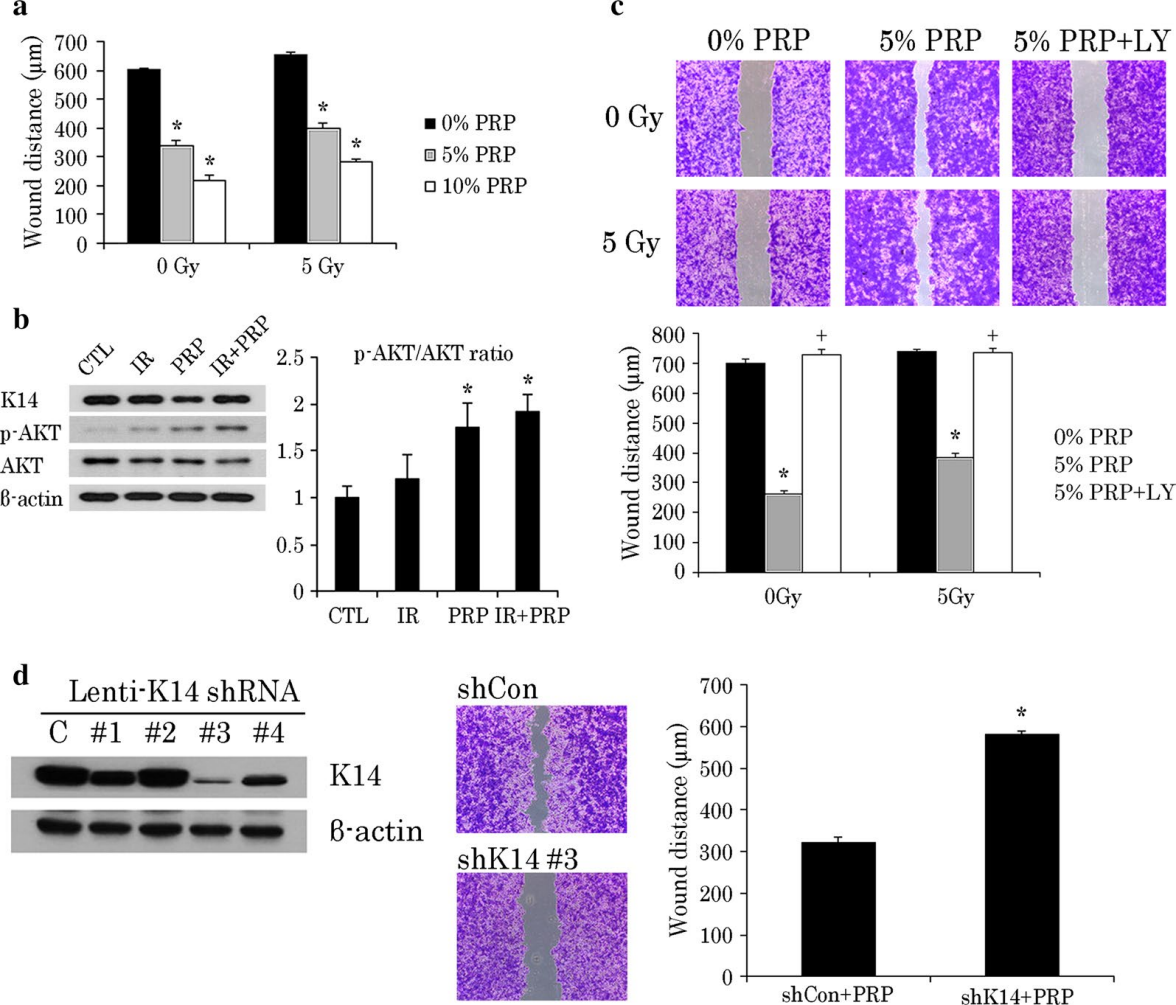
PRP promotes wound healing of irradiated HaCaT cells via AKT signaling. **a** Comparative wound-healing capacity of HaCaT cells at different doses of PRP with or without IR, using scratch-simulated wound migration assay. Result of one experiment performed in triplicate and expressed as mean ± SEM (Student’s *t*-test applied) *p < 0.05 vs. 0% PRP group. **b** Western blot analysis of K14, p-AKT, and AKT expression levels in 5 Gy irradiated HaCaT cells incubated with 5% PRP for 24 h. Quantification of p-AKT/ATK ratio expressed as mean ± SEM (Student’s *t*-test applied) *p < 0.05 vs. IR group. **c** Wound-healing capacity of HaCaT cells incubated with 5% PRP and in presence or absence of AKT signaling blocker, LY294002. Experiments performed in triplicate and repeated three times. Results of one experiment shown expressed as mean ± SEM (Student’s *t*-test applied) *p < 0.05 vs. 0% PRP group and ^+^*p*<0.05 vs. 5% PRP group; and **d** effect of PRP on wound healing in HaCaT cells with or without K14 knockdown. Results of one experiment performed in triplicate and expressed as mean ± SEM (Student’s *t*-test applied) *p < 0.05 vs. shCon group. *IR* irradiation, *CTL* control, *PRP* platelet-rich plasma

In injured skin, epidermal wound healing is regulated by K14-positive cells [12]. Given that keratin expression changes at IR doses beyond 5 Gy (Fig. 1c), we used a 5-Gy dose in examining the wound-healing capacity of irradiated HaCaT cells. As a result, K14 protein levels were sustained, but the growth and migration of irradiated HaCaT cells were significantly impaired (Fig. 1d). On the other hand, both functions recovered substantially through PRP administration (Fig. 2a).

### PRP activated AKT signaling in a K14-dependent manner

At this point, the mechanism by which PRP restored growth/migration of irradiated HaCaT cells was still in question. According to a recent publication, K14 may modulate the PI3K pathway, prompting phosphorylation at the serine 473 residue of AKT [12]. In vitro and in vivo studies have shown that the PI3K/AKT pathway is canonical in proliferating epithelial cells [25]. Thus, we presumed that in HaCaT cells, PRP-induced growth/migration would hinge on AKT signaling. Curiously, PRP increased AKT phosphorylation in both normal keratinocytes and irradiated cells (Fig. 2b). However, the scratch healing promoted by PRP in HaCaT cells was attenuated by AKT inhibition (Fig. 2c). K14 expression was silenced using lentiviral K14 shRNA in HaCaT cells, as proven by western blots. The #3 lentivirus shows the highest efficiency to silence K14 levels. Therefore the #3 K14 knockdown cells were used for the wound-healing capacity. The scratch-simulated wound migration assay revealed that K14 knockdown inhibits migration of HaCaT cells after PRP treatment (Fig. 2d). Taken together, these outcomes implicate K14 in PRP-induced AKT phosphorylation and the wound-healing capacity of irradiated HaCaT cells.

### PRP accelerated wound closure of irradiated skin in irradiated mice

**Fig. 3:**
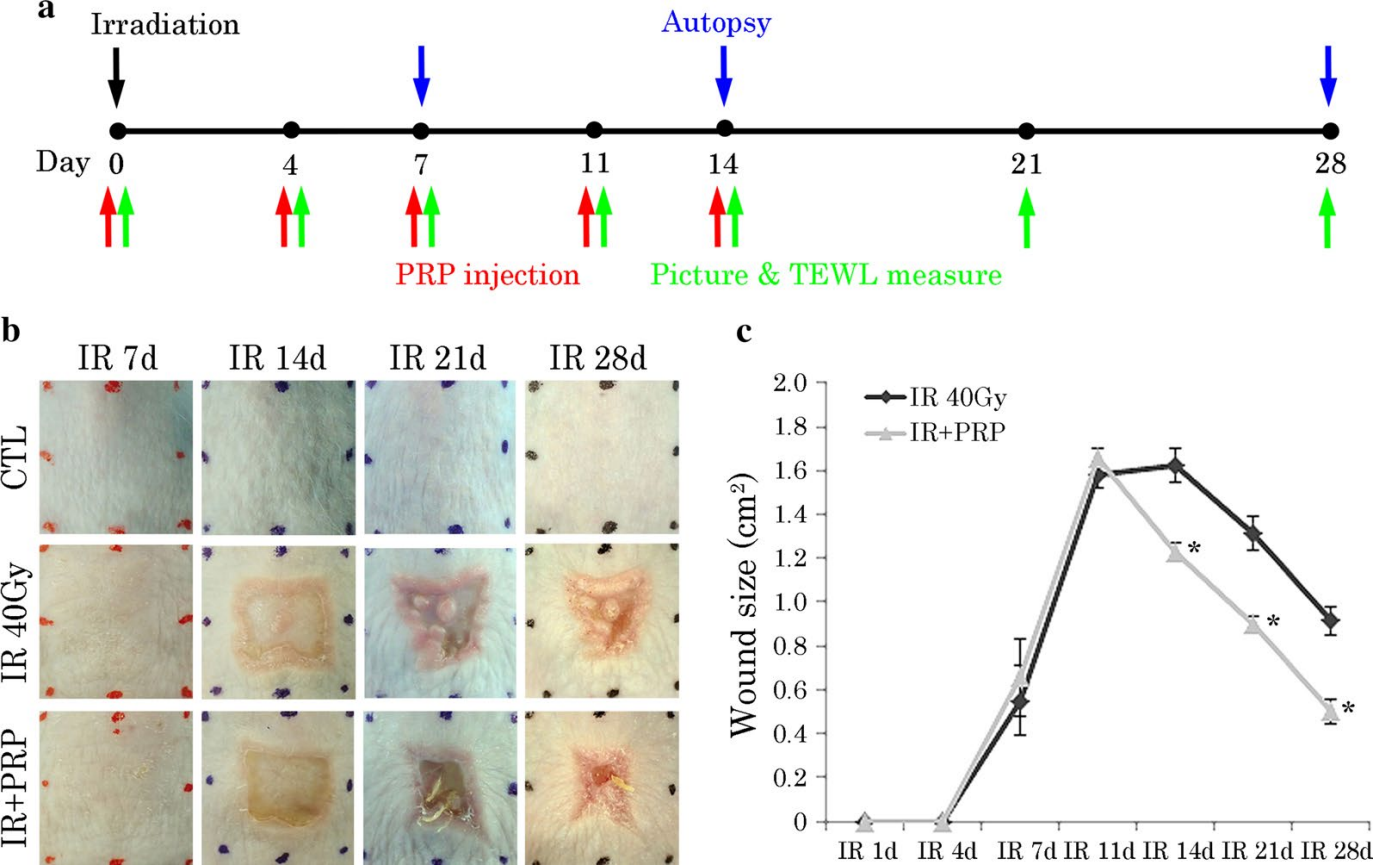
Progression of wound healing in radiation skin-injury mouse model. **a** Schematic illustration of the in vivo assessment. **b** Gross images of changes in mouse skin by group after IR, day 7–28; and **c** quantification of wound size expressed as mean ± SEM (Student’s *t*-test applied) *p < 0.05 vs. 40-Gy group. *IR* irradiation, *CTL* control, *PRP* platelet-rich plasma

To verify the effects of PRP on regeneration of radiation-induced skin injury, we invoked an irradiated skin-wound model in mice treated with PRP. Areas of irradiation were locally injected with either a 0.9% NaCl solution (IR group) or 100 µl PRP (PRP group). No adverse effects were observed at any time during the experimental procedure (Fig. 3b). Digital photographs chronicled the progress made in wound resolution by IR and PRP groups. All wounds routinely contracted over time, but wound size in PRP-treated mice was less than that in IR, and unlike the open wounds of IR mice, had nearly closed at day 28. Quantification of active wound dimensions confirmed significantly faster resolution of wounds in the PRP (vs. IR) group at all time points (Fig. 3c). These results underscored the benefits of locally injected PRP in terms of promoting the regeneration of irradiated skin.

**Fig. 4:**
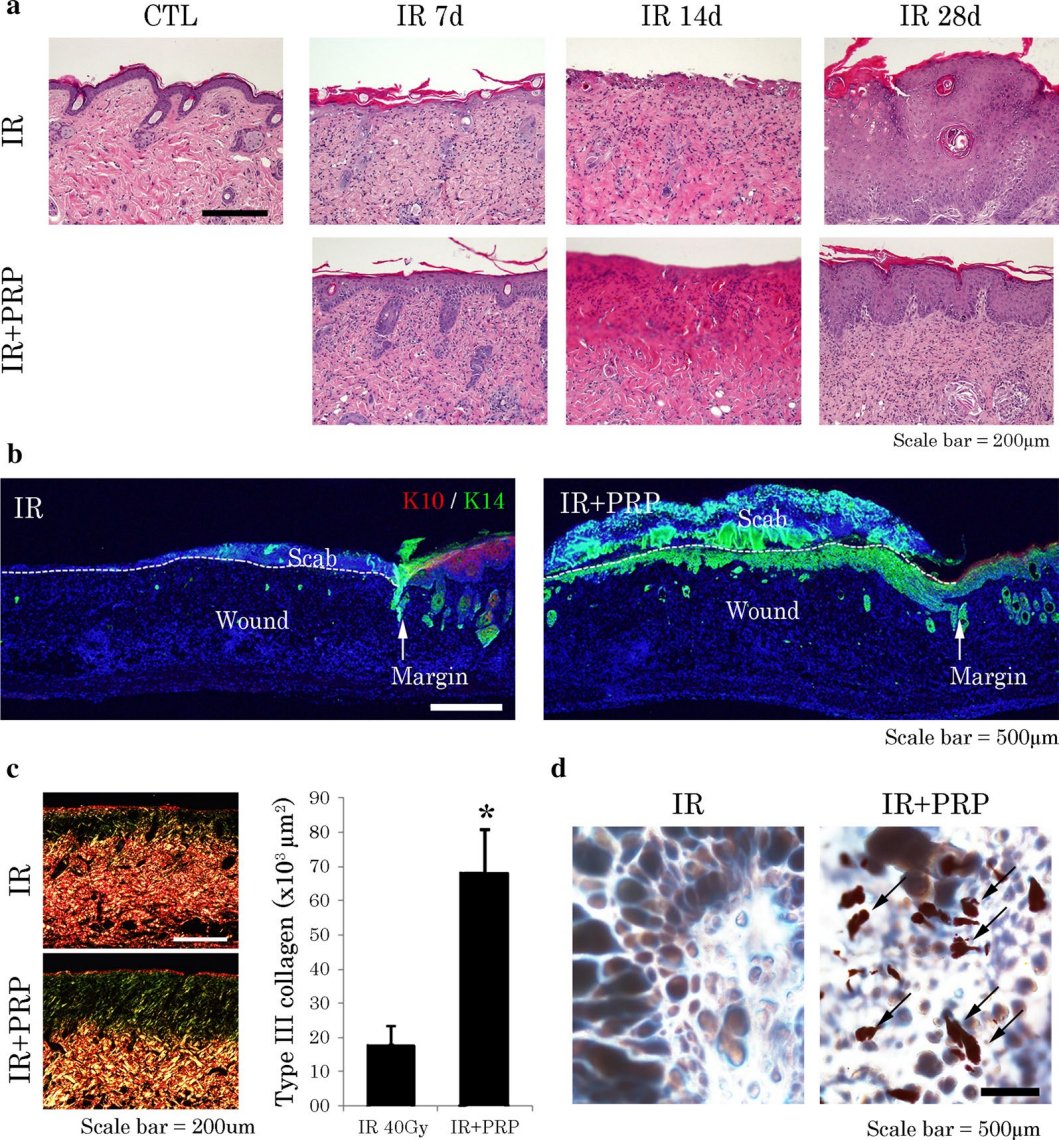
Histologic features of radiation skin-injury mouse model. **a** Comparative images of PRP-treated and untreated irradiated skin (H&E stain) at day 7, 14, and 28. **b** K10/K14 positivity on immunostain (note epithelial tongues) in irradiated skin at day 14. **c** Quantification of type III collagen in irradiated skin at day 14 (Picrosirius Red stain). Results of one experiment performed in triplicate and expressed as mean ± SEM *p < 0.05 vs. 40-Gy group; and **d** gland-like structures (arrows) encountered in recovering skin at day 28 (Oil Red O stain). *IR* irradiation, *CTL* control; *PRP* platelet-rich plasma

The extent of skin regeneration was also assessed microscopically. Photomicrographs of histologic preparations indicated that after PRP treatment, epidermal thickness and layering were more aligned within normal skin at day 28, surpassing the IR group in this regard (Fig. 4a). In H&E and Sirius red-stained sections, group-wise differences in the efficiency of repair were demonstrable at day 14 (Fig. 4a, c). In treated wounds, type 3 collagen fibers atop wound beds were more plentiful, indicating enhanced granulation tissue formation and epithelial regeneration. K14-expressing epithelial tongues were also observed beneath eschar (scabs) in the PRP group (Fig. 4b), and there were more constructs resembling sebaceous glands in defects of PRP treated (vs untreated) mice at day 28 (Fig. 4d).

### PRP enhanced Keratin 14 expression and promoted keratinocyte proliferation in irradiated mice

**Fig. 5:**
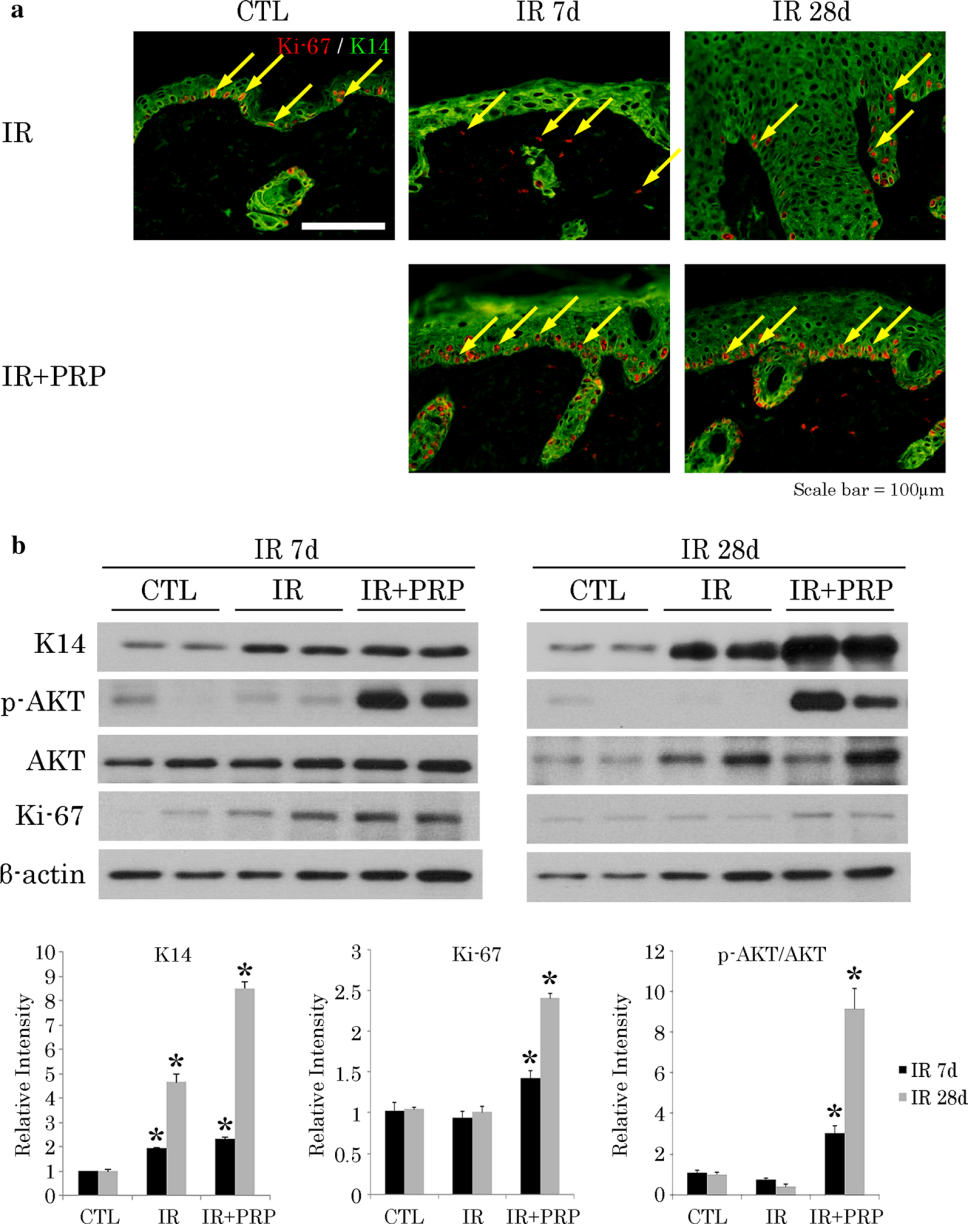
Proliferation of K14-positive cells in radiation skin-injury mouse model. **a** Ki-67 positivity (arrows) on immunostain; and **b** Western blot analysis of p-AKT, AKT, and Ki-67 expression levels at wound sites. Results of one experiment performed in triplicate and expressed as mean ± SEM *p < 0.05 vs. IR group. *IR* irradiation, *CTL* control, *PRP* platelet-rich plasma

**Fig. 6:**
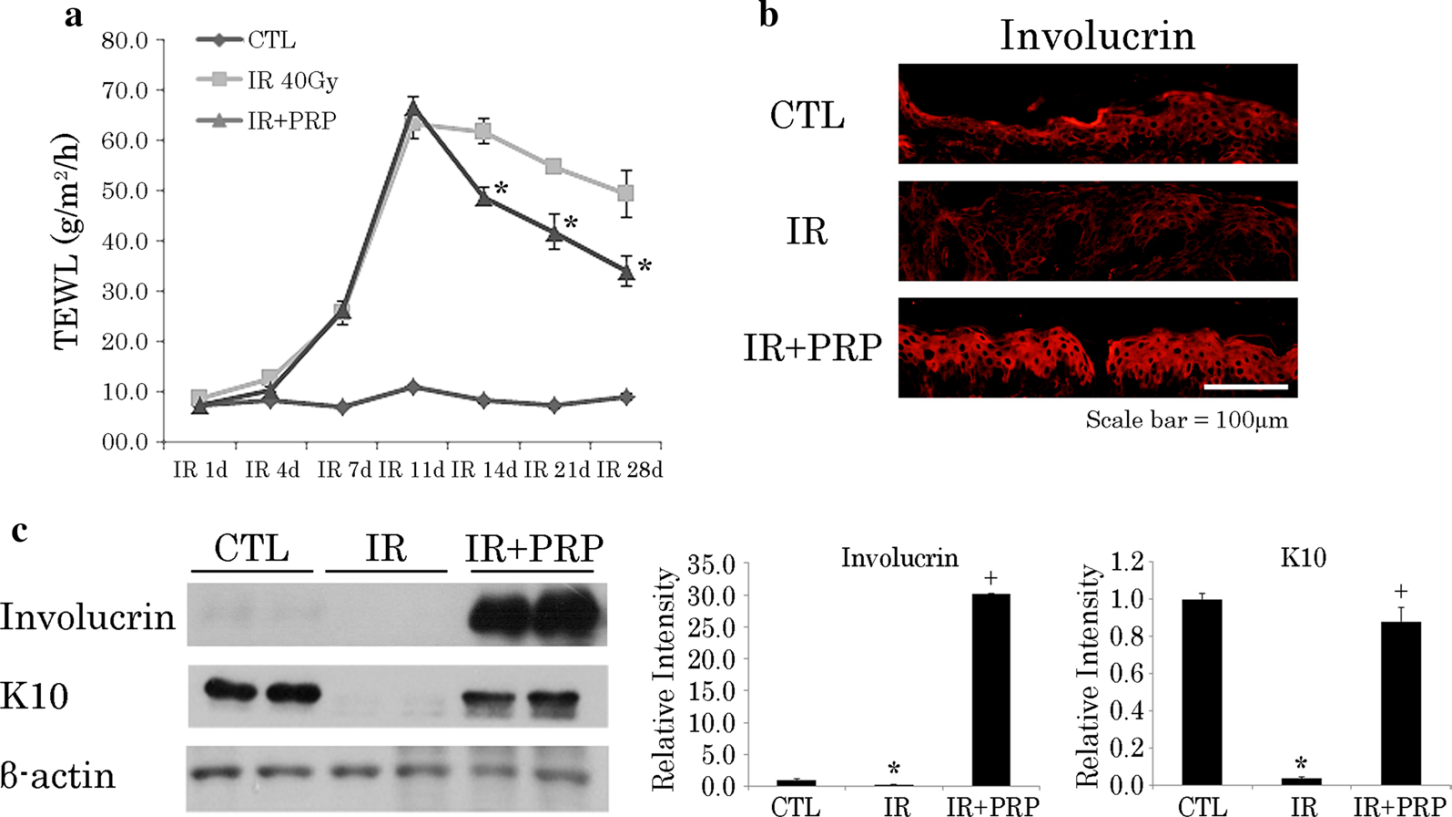
Fluctuating cutaneous barrier function in radiation skin-injury mouse model. **a** Chronology of transepidermal water loss in recovering irradiated skin. Data are expressed as mean ± SEM *p < 0.05 vs. 40-Gy group; and **b** involucrin positivity on immunostain in recovering skin at day 28. **c** Western blot analysis of Involucrin and K10 expression levels in recovering skin at day 28. Results of one experiment performed in triplicate and expressed as mean ± SEM *p < 0.05 vs. CTL group, ^+^*p*<0.05 vs. IR group. *IR* irradiation, *CTL* control, *PRP* platelet-rich plasma

Our next focus was assessment of K14 and Ki-67 expression levels in irradiated skin samples, proving the importance of K14 in epithelial regeneration. Immunostaining of K14 and Ki-67 provided insight into the cellular proliferative capacity within epithelial layers (Fig. 5a). Both K14 and Ki-67 were significantly upregulated in skin of PRP-treated mice, compared with IR (Fig. 5b). We then attempted to verify the mechanism involved in PRP-related upregulation of K14. Western blotting was used to determine AKT and p-AKT expression levels in irradiated skin after PRP treatment. Compared with the IR group, PRP treatment conferred a significant increase in phosphorylated AKT (Fig. 5b), indicating that PI3K/AKT signaling was indeed activated by PRP. Expression of K10 also increased in the PRP group, confined to suprabasal cells (Fig. 6c). These results affirm the cutaneous recuperative effects of PRP treatment.

### PRP enhanced skin barrier function in irradiated skin wound

Because K10-expressing cells have a role in the barrier function of skin, we pursued this avenue of investigation as well. TEWL is a physiologic property reflecting skin barrier efficiency. TEWL measurements allowed us to compare the reconstitution of barrier function and maturation of wounds in PRP-treated and untreated mice over time. Despite inevitable wound contraction, TEWL values in the PRP group were less than those of the CTL group. Specifically, the PRP group showed a nearly linear decline in TEWL values as of day 14, whereas values in the CTL group had not fallen significantly (Fig. 6a). Finally, we evaluated the expressions of K10 and involucrin in irradiated skin samples to verify barrier function repair. K10 and involucrin were upregulated in epithelial layers of PRP-treated (vs untreated) mice (Fig. 6b, c).

## Discussion

Improving endogenous basal keratinocytes and their inherent functions is a key factor in skin repair, especially under suboptimal conditions. In the course of this study, we discovered that unlike normal controls, keratinocytes from radiogenic wounds were marked by shifts in keratin expression (K10 to K14 keratin); and in immunofluorescent analysis, K14 expression broadened to include suprabasal cells. These revelations were further confirmed in irradiated HaCaT cells. In addition, the proliferation and migration of such altered keratinocytes seemed to be impaired. K14 is typically found in the basal layer where it serves to control the proliferation of cells. Previous reports have shown that K14 is explicitly expressed in the mitotically active basal layer of stratified epithelia, wherein epidermal stem cells and TA cells reside [26]. In another in vitro study, primary cultures developed from K14-null mice showed a reduced capacity for cellular proliferation [27]. However, our results indicate that K14 in irradiated keratinocytes hampered the ability to participate in skin repair. Consequently, restoration of functional K14 in keratinocytes may be essential for adequate repair of irradiated skin.

Clinical applications intended to functionally activate endogenous basal cells are part of an advanced strategy adopted by regenerative medicine and tissue engineering for reduction of risk. Since the 1970s, PRP has been a familiar clinical commodity, recognized for its regenerative and healing properties [28–31]. It has been shown to enhance the proliferation and differentiation of cells, primarily through a variety of growth factors secreted by platelets (i.e., PDGF-AB, TGF-β, and FGF) [32–34]. In a previous study of ours, we have proven that our method of PRP activation yields a more concentrated release of growth factors for superior growth and migration of mesenchymal stromal cells (MSCs), compared with traditional methods of PRP activation^18^. Thus, we launched this investigation of radiation-induced skin damage, hoping to improve the reparative process through activated PRP application.

To assess the regenerative effects of PRP on irradiated skin, we examined the wound healing capacity of irradiated HaCaT cells incubated with PRP. According to one in vitro study, PRP treatment promotes wound healing properties and activates AKT signaling in irradiated HaCaT cells. These particular effects are also blocked by AKT inhibitor treatment. Activation of PI3K/AKT signaling has been viewed as the chief mechanism driving proliferation and migration of K14 cells [12]. As recent reports indicate, PRP may induce PI3K/AKT signaling, serving to enhance survival and regenerative functions of MSCs [22, 35]. To further explore the relation between PRP on K14-positive cells, we used shRNA to create a K14-knockdown HaCaT cell line, which readily displayed inhibition of PRP-induced wound healing properties.

To validate the wound-healing effects of PRP on irradiated skin, we used a preclinical model of radiation-induced skin injury in mice. Through clinical observations, histopathologic analysis, and functional evaluation of the skin barrier, we have ascertained that local injection of PRP clearly promotes cutaneous wound healing. Microscopic examination of irradiated mouse skin injected with PRP revealed an lush mantle of granulation tissue, including new and thicker type III collagen and epithelial tongues composed of K14-positive cells, all materializing within 14 days. At day 28, structures resembling sebaceous glands were also observed in PRP-treated skin.

When proliferating keratinocytes in irradiated lesions were subsequently immunostained, focusing on Ki-67 expression and AKT signaling activation, Ki-67-positive cells were frequent in regenerative PRP-treated skin, compared with controls; and results were analogous for AKT signaling. Finally, the diminished expression of K10 we observed after irradiation was reversed through PRP treatment. K10 is expressed in the suprabasal layer of skin, and in K10-deficient mice, the permeability barrier function is diminished [36]. Reduced K10 expression leads to changes in expression levels of cornified envelope proteins, which form a scaffold for permeability barrier purposes [36, 37]. Expression of involucrin, a cornified envelope protein, increased in PRP-treated irradiated skin, and heightened TEWL values were reversed. Overall, these findings demonstrate that PRP enhances the proliferation of K14 cells and accelerates cutaneous barrier restoration in a mouse model of radiation-induced skin injury.

## Conclusion

The present efforts indicate a shift from K1/10 to K14 status in irradiated keratinocytes and demonstrable impairment of wound healing. However, PRP acts to reverse radiation injury via AKT signaling, thus enhancing the wound-healing and barrier functions of skin. PRP may therefore be clinically beneficial in this sphere, helping to overcome poor cutaneous healing after radiation exposure.

## Data Availability

Please contact author for data requests.
